# Ornithogenic vegetation: How significant has the seabird influence been on the Aleutian Island vegetation during the Holocene?

**DOI:** 10.1002/ece3.8121

**Published:** 2021-09-14

**Authors:** Olesya Igorevna Smyshlyaeva, Elena Erastovna Severova, Olga Aleksandrovna Krylovich, Evgeniya Andreevna Kuzmicheva, Arkady Borisovich Savinetsky, West Dixie, Virginia Hatfield

**Affiliations:** ^1^ Laboratory of Historical Ecology Severtsov Institute of Ecology and Evolution Russian Academy of Sciences Moscow Russia; ^2^ Biology Faculty Department of Higher Plants Lomonosov Moscow State University Moscow Russia; ^3^ Faculty of Biology and Biotechnologies National Research University Higher School of Economics Moscow Russia; ^4^ Biodiversity Institute University of Kansas Lawrence KS USA; ^5^ Museum of the Aleutians Unalaska AK USA

**Keywords:** Aleutian Islands, islands ecosystems, pollen analysis, seabird guano, vegetation dynamics

## Abstract

In the Aleutian Islands during the Holocene, terrestrial predators were actually absent; as a result, large seabird colonies thrived along the coasts or across entire islands. Bird guano enriches the soil with nitrogen, which can lead to the formation of highly modified ornithogenic (bird‐formed) ecosystems. For a more detailed investigation of avian influence, we reconstructed more than 10,000‐year‐old vegetation dynamics of the coast of Shemya Island (Near Islands) by pollen analysis. At the initial stages of vegetation development (10,000–4,600 cal year BP), sedge–heather tundra grew in the studied area. A seabird colony existed on Shemya from 4,600 to 2,400 cal year BP according to stable isotope analysis. During a period of at least 2,200 years, nitrogen enrichment led to the development of ornithogenic herb meadows with a high presence of Apiaceae. A long‐term increase in δ15N above 9–10‰ led to radical shifts in vegetation. Noticeable reduction of seabird colonies due to human hunting led to grass‐meadows spreading. After a prolonged decrease δ15N below 9–10‰ (2,400 cal year BP to present), there was a shift toward less productive sedge‐tundra communities. However, the significant enrichment of guano affected only the coastal vegetation and did not alter the inland Shemya Island.

## Graphical abstract

On the Aleutian Islands, seabird colonies can have significant impacts on vegetation, as well as climate and volcanic eruptions. We reconstructed the dynamics of vegetation, which was formed under the influence of bird guano by pollen analysis. Long‐term influences drastically change communities and pollen spectra.

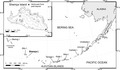

## 1 INTRODUCTION

Vegetation history on the oceanic islands is one of the crucial points of island ecology. Plant community dynamics can be affected by a variety of factors; changing climatic conditions is one of most important (Bennett et al., 1997; Bunting, 1994; Lawson et al., 2005; Molloy & O’Connell, 2004; Weigelt et al., 2016). Besides climate, there are a lot of other drivers as well as altitude, area and geological age of islands, soils, oceanic flows, isolation, volcanic activity, human activity, etc. (Garroutte et al., 2018; Heusser, 1990; Lawson et al., 2008; McCord, 1980). According to their geography and geology, the Aleutian Islands serve as a model for observing the vegetation history during the Holocene.

The Aleutian Islands are a volcanic origin archipelago extending almost 2,000 km from Kamchatka to mainland Alaska. The fundamental climatic patterns of the Aleutian Islands are determined by the position of the Aleutian Low (Broadman et al., 2020; Rodionov et al., 2005, 2007). Mild winters, high humidity, cloudiness, fogs, frequent storms, and strong winds are the features that define the Aleutian landscape (Hultén, 1968). The proximity of Kamchatka to the west of the Aleutian arc and Alaska to the east also enhances the difference in the flora and vegetation between the western and eastern islands (Garroutte et al., 2018; Garroutte & Ickert‐Bond, 2013; Hulten, 1937). There are only 520 vascular plant species across the arc, and species richness of plant communities is less than in the same communities on the mainland; therefore, these ecosystems are very sensitive to various disturbances (Garroutte, 2018). According to vegetation studies, the plant communities also depend on altitude, drainage, and soil properties (Byrd, 1984; Hulten, 1968; Talbot et al., 2010; Talbot & Talbot, 1994). Another important factor is the snow distribution in winter, which is determined by the wind strength and topography (Hultén, 1937, 1968). At the same time, the islands are located practically at the same latitude, which is why they are unique model objects for studying the interaction and influence of all the above driving forces.

Regional flora and vegetation were formed mainly in the Holocene (Garroutte et al., 2018; Hulten, 1968). Among more than 200 Aleutian Islands, the Holocene vegetation dynamics have been reconstructed on Attu (Heusser, 1990), Tanaga (Anderson & Bank, 1952), Adak (Heusser, 1978; Noguchi et al., 2018), Atka (Heusser, 1990), Carlisle (Kuzmicheva et al., 2019), Umnak (Heusser, 1973), and Unalaska (Anderson & Bank, 1952). In these studies, climate was considered as the main factor affecting vegetation, but it was difficult to identify the general pattern of plant communities changing. The most noticeable shifts were in dominance between herbaceous taxa and dwarf shrub or between sedges and grasses as a result of changes in temperature and/or moisture. However, along the entire archipelago, the shifts happened asynchronously and occasionally did not coincide with the main climatic phases. These inconsistencies, among other things, are also associated with strong volcanic activity in this region. Eruptions and ash falls are the second most important factor determining the landscape and vegetation of the Aleutian Islands in the Holocene (Heusser, 1990; Kuzmicheva et al., 2019; Noguchi et al., 2018). As a result of eruptions, plant communities are sometimes completely disrupted, and their formation begins anew (Talbot et al., 2010). Ashes alter the chemical composition of soils, which also affects ecosystems (Kuzmicheva et al., 2019). Nevertheless, it also remains unknown how exactly volcanic activity influenced vegetation over long‐term timescales.

When reconstructing the dynamics of Aleutian Islands plant communities, researchers focused on the above two factors (Heusser, 1973, 1978, 1990; Kuzmicheva et al., 2019; Noguchi et al., 2018). Seabird activity as an important factor remains insufficiently studied. The Aleutian Islands are remarkable because there were predominantly no terrestrial predators east of the Commander Islands and west of Umnak prior to the start of their human colonization in 1741 (Crockford, 2012; Maron et al., 2006; West et al., 2007). Due to this, billions of birds have formed large colonies on the Aleutian Islands over thousands of years (Byrd, Day&, 1986; Crockford, 2012; Croll et al., 2005; Krylovich et al., 2019; Maron et al., 2006). According to botanical investigations on other North Pacific Islands, even short‐term impact of seabird colonies lead to changes in vegetation cover, and in the soil chemistry or bedrock and eventually to the formation of ornithogenic ecosystems and vegetation (Ivanov, 2013). The long‐term effects of birds and dynamics of their colonies during the Holocene have been studied in Greenland and Svalbard by the stable isotope analysis of lake sediments and peat cores (Davidson et al., 2018; Gąsiorowski & Sienkiewicz, 2019; Yuan et al., 2010). Seabirds provide large amounts of marine organic matter to nutrient‐limited terrestrial ecosystems by guano (Caut et al., 2012; Maron et al., 2006), which is reflected in the sediments by the enrichment of the heavy nitrogen isotope, namely significant increase of δ^15^N value (Croll et al., 2005; Davidson et al., 2018; Gąsiorowski & Sienkiewicz, 2019; Maron et al., 2006; Szpak et al., 2012; Yuan et al., 2010). We hypothesized that for Aleutian Islands plant communities depleted by abundant rainfalls and intense leaching, this fertilization has a significant impact. However, it remains unknown whether vegetation changes under the influence of millennial‐scale dynamics of seabird colonies.

At the same time, in the Aleutian Islands, relatively short declines in seabird impact on ecosystems have already been studied. On islands, where Arctic foxes (*Vulpes lagopus*) have been introduced since the 18th century, plant communities have changed from highly productive grasslands to dwarf shrub‐dominated ecosystems (Croll et al., 2005; Maron et al., 2006). There was a significant difference in the δ^15^N values of soils and plants between fox‐free and fox‐infested islands, although this distinction could vary greatly depending on the island size and the distance from the coast. The δ15N value increased up to 14‰ in soils and almost 16‰ in grasses on the fox‐free islands (Maron et al., 2006). Based on these results, the guano influence on the coastal ecosystem of Carlisle Island (Islands of Four Mountains, Aleutian Islands) during the Holocene was found by the isotopic signature (Kuzmicheva et al., 2019). However, noticeable shifts in vegetation were not detected due to the earlier peopling of the eastern part of the Aleutian Islands, the short‐term impact, and many ash layers in the peat core (Kuzmicheva et al., 2019). Higher δ15N values over a longer period of time were found in an ash‐free coastal peat deposit on Shemya Island, one of the westernmost islands of the archipelago (Savinetsky et al., 2014). Compared to the inland peat core of this island, the values of the heavy nitrogen isotope were several times higher during most of the Holocene (Savinetsky et al., 2010, 2014). According to archaeological data, the first settlements appeared on Shemya Island about 3,000 cal year BP, which coincides with the time when the δ15N value in the coastal deposit decreased (Lefevre et al., 2010; Savinetsky et al., 2014). The existence and subsequent significant reduction of the colony is also confirmed by zooarchaeological discoveries in archaeological sites (Lefèvre et al., 2010). We hypothesized that, in contrast to the inner vegetation (Smyshlyaeva et al., 2021), the coastal one reflected not only climate impact but also the considerable seabird nutrient input.

To study the seabird impact on Aleutian Islands plant communities, we reconstructed vegetation dynamics recovered from the coastal peat deposit (McDonald Point) on Shemya Island using pollen analysis. We investigated the composition of this profile by radiocarbon dating, age‐depth model and estimation of sediment rate, and loss‐of‐ignition (LOI) to more accurately interpret vegetation history. In this paper, we answer two questions: Do long periods of intensive seabird impact affect coastal Aleutian Island vegetation? And how do plant communities change after reduction of these impacts?

## 2 MATERIALS AND METHODS

### 2.1 Sampling

**FIGURE 1:**
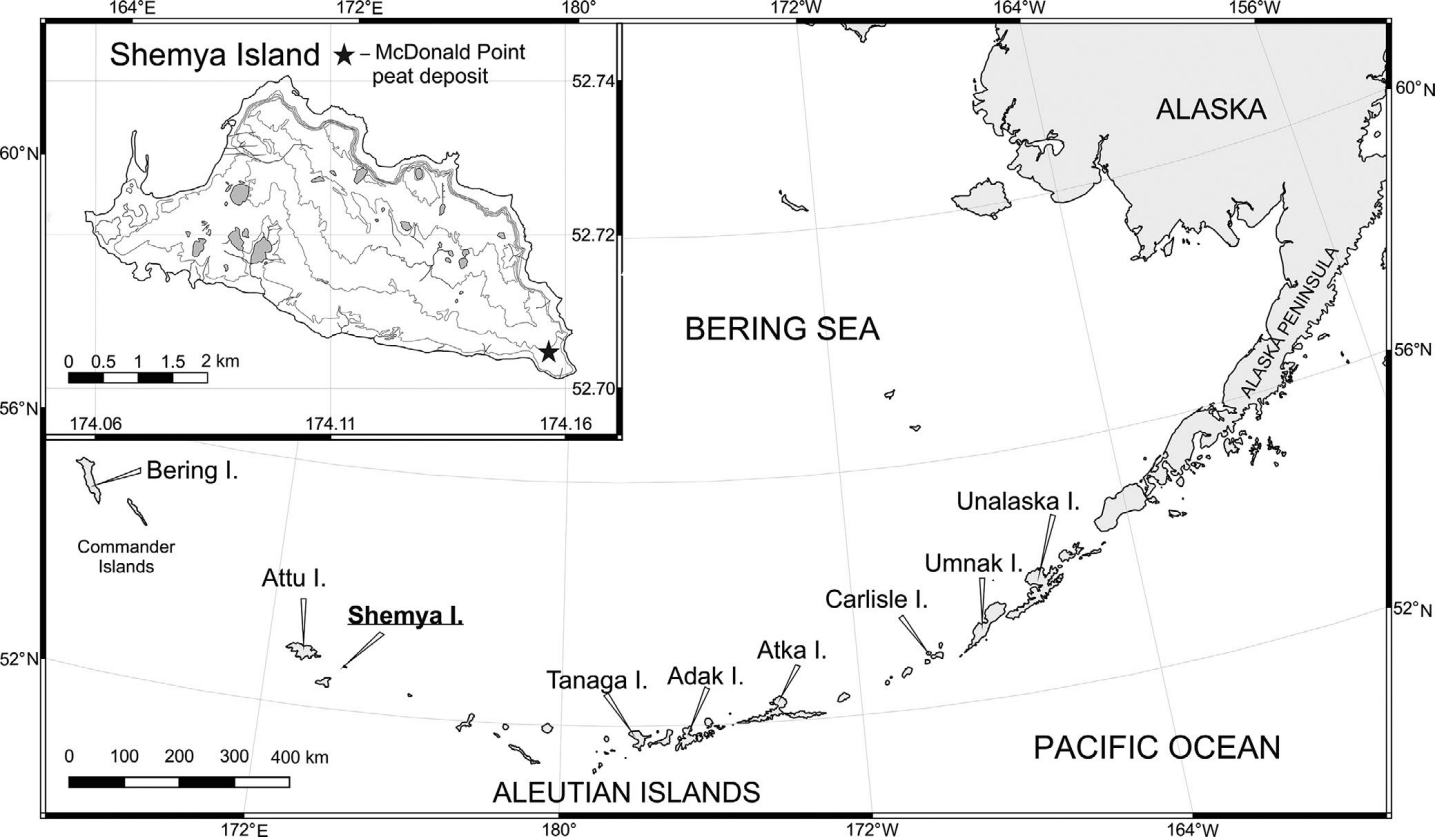
The study area map. The inset shows Shemya Island (the Near Islands) and the McDonald Point peat deposit (mark)

Shemya is a flat island, devoid of volcanoes, located at 52°N and 174°E. It is approximately 4.5 km wide by 7 km long with a total area of 15 km² (Figure 1). The island relief is gently hilly. The vegetation is represented by two types of communities, with herbaceous plant and dwarf shrub dominant (Causey et al., 2010; Kiseleva et al., 2002; Savinetsky et al., 2014). Grasses and sedges, along with a noticeable abundance of forbs, dominate the stream valleys, lowlands, and slopes of different exposures. These communities are more diverse on well‐drained southern slopes. Meadow vegetation dominated by *Leymus mollis*, *Heracleum lanatum*, and *Senecio pseudo‐arnica* occurs on hills near the sea coast (Savinetsky et al., 2010). Sedges often become dominant under waterlogged conditions (Causey et al., 2010; Kiseleva et al., 2002). The second type of community is dominated by dwarf shrubs (*Empetrum nigrum*, *Vaccinium vitis‐idea*, *Linnea borealis*) and occurs on more exposed sites (Causey et al., 2010; Kiseleva et al., 2002).

A peat deposit was sampled at McDonald point on the east coast of Shemya Island in 1999 (Figure 1). The land surface was flat, slightly sloping, with a steep ledge protruding into the sea. The ledge was cut during quarrying and exposed the bedrock and peat profile. The depth of the sampled core was 385 cm. We cleaned and smoothed out the exposure. Samples were taken into plastic bags by a trowel layer by layer; their thickness was on average 5 cm, taking into account the sediment stratigraphy. The trowel was cleaned after each sample. All samples were taken to the laboratory and stored in a cold room.

#### 2.1.1 Radiocarbon dating

**FIGURE 2:**
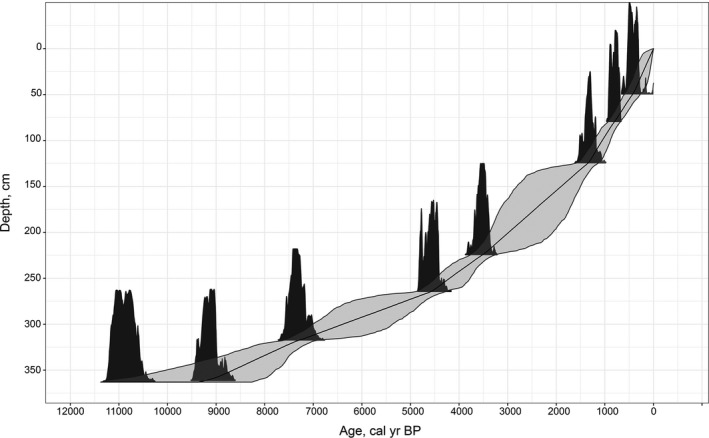
McDonald Point age‐depth model. Dark gray— calibrated dates, gray—95% chronology confidence interval

To determine the chronology and accumulation rate of the sediment, we selected all plant remains from 7 peat samples after acid–alkaline–acid extraction for radiocarbon dating (Table 1). Dating was carried out by the scintillation method (Piotrowska et al., 2011) in the Laboratory of Historical Ecology of A.N. Severtsov Institute of Ecology and Evolution of Russian Academy of Sciences. Radiocarbon dates were calibrated to determine calendar ages and to frame the age‐depth model (Figure 2) by the “IntCal13” ground calibration curve (Reimer et al., 2013). We carried out these calculations and sedimentation rate in the package “Bchron” 4.2.6 (Haslett & Parnell, 2008; Parnell, 2015) in the statistical environment R v. 3.6.2 (R Core Team, 2019). Calibrated dates are given in the text below.

**TABLE 1:** ^14^C ages of McDonald Point peat deposit

Depth (cm)	^14^C age (year BP)	Age range (cal year BP) 2*σ*	Median age (cal year BP)	Lab ID
50–55	400 ± 100	614–159	425	IEMAE−1243
80–85	875 ± 65	918–692	796	IEMAE−1285
125–130	1,405 ± 100	1520–1094	1,323	IEMAE−1280
225–230	3,295 ± 90	3772–3343	3,528	IEMAE−1279
265–270	4,060 ± 80	4811–4326	4,569	IEMAE−1262
318–320	6,430 ± 155	7580–6977	7,335	IEMAE−1286
363–372	9,550 ± 130	11185–10504	10,879	IEMAE−1261

#### 2.1.2 Loss‐of‐ignition

To determine organic content dynamics, a total of 65 samples were placed in the crucible and then in the muffle furnace at 550°C for at least 2 hr for measuring organic content. To calculate LOI values, we subtracted weight after ashing from the dry weight and expressed it as % (Chambers et al., 2010).

#### 2.1.3 Pollen analysis

To reconstruct vegetation dynamics, a total of 66 samples 2–4 cm^3^ in size with an interval of 5–10 cm were selected for pollen analysis. We used boiling in 10% HCl, 10% KOH, washing through a sieve (250 µ), boiling in HF, acetolysis, according to the standard procedure with some modifications for chemical processing of samples (Chambers et al., 2011; Faegri & Iversen, 1975). To determine the concentration of pollen and spores, the special markers were added before chemical treatment (Stockmarr, 1973). We added two tablets of *Lycopodium clavatum* spores from batches #483216 or #938934 to each sample. Pollen atlases and keys (Reille, 1998, 1999), electronic databases (https://globalpollenproject.org), and a reference collection (Severova et al., 2016; http://botany‐collection.bio.msu.ru) were used to determine pollen and spores taxa.

Using an Axioskop ZEISS light microscope with a magnification of ×400, we counted pollen up to at least 500 pollen grains in those samples, where possible. The percentage of pollen types was calculated from the total of all pollen in the sample, and the percentage of spores from the total of pollen and spores. Total pollen influx was calculated by multiplying the total pollen concentration and sedimentation rate of each sample (Hicks & Hyvärinen, 1999). Tilia software v.2.0.41 was used to construct pollen diagrams (Grimm, 2015). Percentage and concentration diagrams are presented in abbreviated form; taxa that occur singly are excluded. Complete diagrams and spreadsheets are available in the Data Accessibility Statement. We identified pollen zones by stratigraphically constrained cluster analysis (CONISS) (Grimm, 2015).

## 3 RESULTS

**FIGURE 3:**
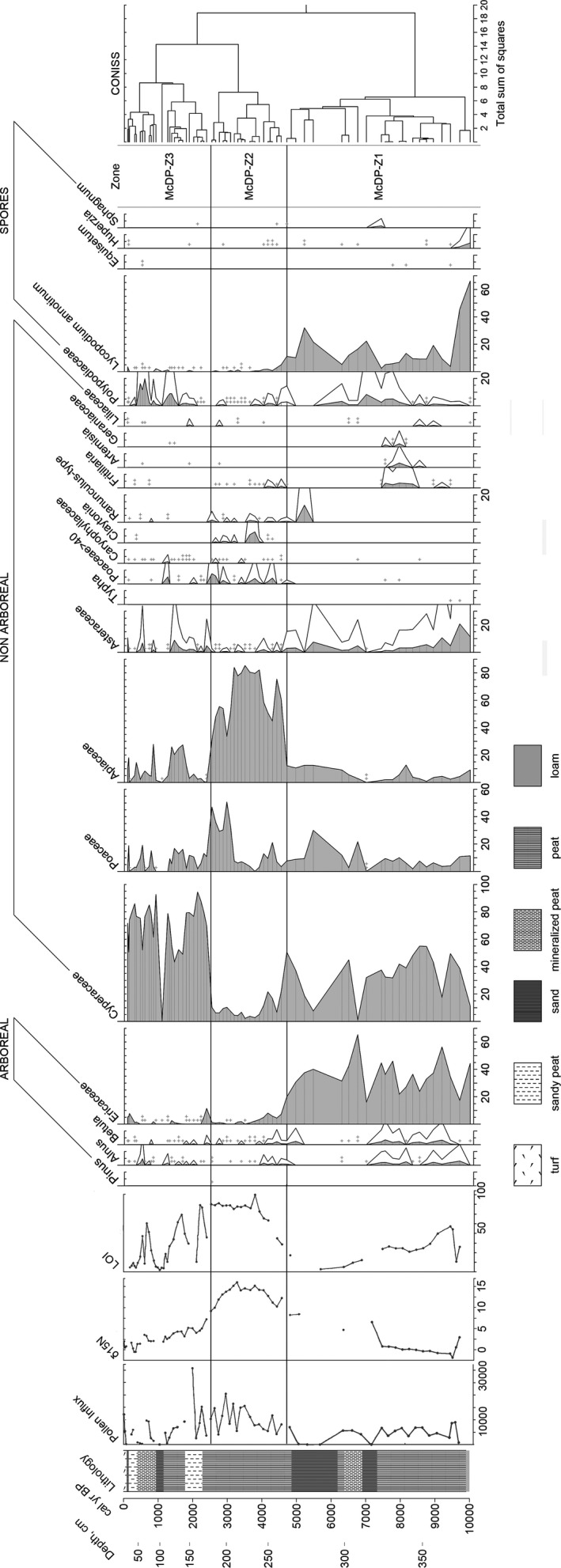
Abbreviated pollen percentage diagram for McDonald Point peat deposit. White curves exaggerated x 5. McDP‐Z1, McDP‐Z2, McDP‐Z3 pollen zones identified on the basis of CONISS are shown by solid lines. Signs + and ++ mark single pollen grains. The full diagram is presented in the Data Accessibility Statement. LOI values are presented in %. Pollen influx is presented in grains/cm^2^/years. **Δ**15N values given from Savinetsky et al. (2014) in ‰. Some more samples were added by the same methodology to reduce the previous gaps

**FIGURE 4:**
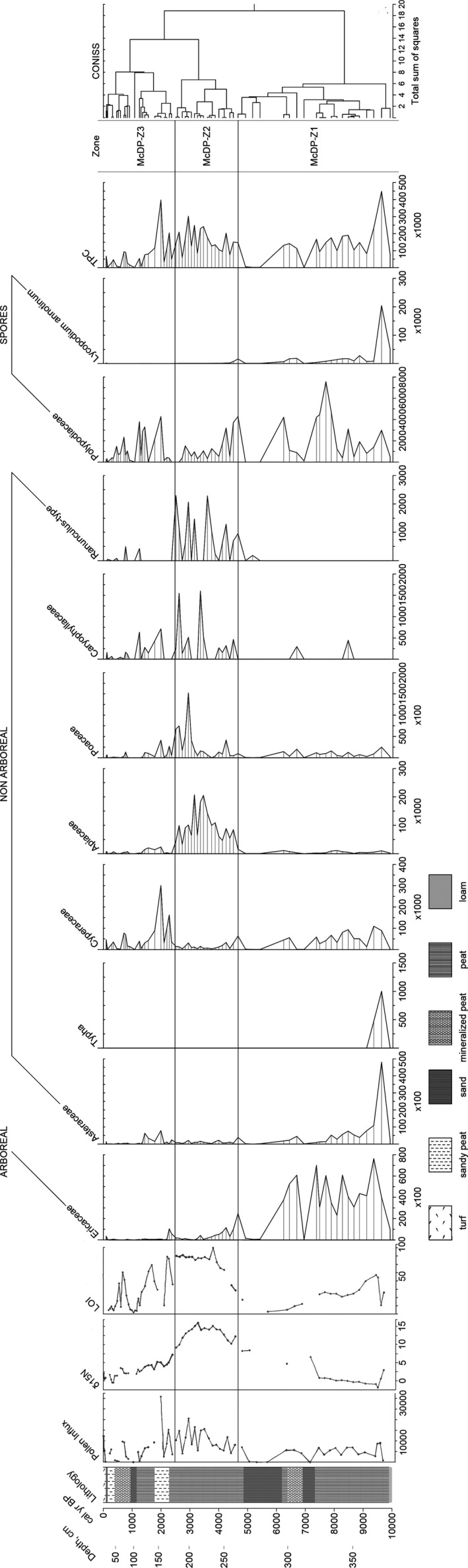
Abbreviated pollen concentration diagram for the McDonald Point peat deposit (grains/cm^3^). McDP‐Z1, McDP‐Z2, McDP‐Z3 pollen zones identified on the basis of CONISS are shown by solid lines. The full diagram is presented in the Data Accessibility Statement. LOI values are presented in %. Pollen influx is presented in grains/cm^2^/years. **Δ**15N values given from Savinetsky et al. (2014) in ‰. Some more samples were added by the same methodology to reduce the previous gaps

The McDonald Point deposit began forming prior to 10,000 cal year BP, as evidenced by the date for 363 cm (Figure 2, Table 1). The sedimentation rate gradually increased to the top of the core from 0.04 to 0.19 cm/year (Figure 2). The LOI was quite low and gradually declined from 10,000 to 5,600 cal year BP, the average was 30%. From 4,750 to 3,750 cal year BP, it gradually increased and then was relatively stable from 3,600 to 2,500 cal year BP, on average 85%. Since 2,500 years ago, the LOI has fluctuated significantly again, averaging 30% (Figures 3 and 4).

In the pollen spectra, we identified a total of 46 palynotypes: 7 arboreal taxa (we included Ericaceae in this group), 31 nonarboreal taxa, and 8 types of spores. This is comparable to recent pollen reconstructions on the Aleutian Islands (Kuzmicheva et al., 2019; Noguchi et al., 2018; Smyshlyaeva et al., 2021) and slightly more than in Heusser's (Heusser, 1973, 1978). The pollen concentration of arboreal taxa was on average 14,600 grains/cm^3^, nonarboreal taxa on average 87,880 grains/cm^3^ and spores on average 9,260 grains/cm^3^. Total pollen influx was on average 6,750 grains/cm^2^/year, which corresponds to amounts in late Quaternary tundra on Atka peat deposit (Heusser, 1990) and other parts of Alaska (Anderson, 1988; Brubaker et al., 1983).

The dominant taxa are Cyperaceae, Apiaceae, Ericaceae, Poaceae, and *Lycopodium annotinum* (Figure 3). The total pollen concentration (TPC) was on average 104,700 grains/cm^3^ (Figure 4), which is higher than the maximum TPC values for Carlisle Island (Kuzmicheva et al., 2019), and significantly higher than in peatlands of Arctic Coastal Plain of Alaska (Eisner et al., 2005).

We recognized 3 pollen zones: McDP‐Z1 (385–265 cm, 10,000–4,600 cal year BP), McDP‐Z2 (265–175 cm, 4,600–2,400 cal year BP), and McDP‐Z3 (175–0 cm, 2,400–0 cal year BP) (Figure 3). Application of CONISS to the pollen concentration data produced the same pollen zonation (Figure 4).

### 3.1 McDonald Point‐1 (385–265 cm, 10,000–4,600 cal year BP)

Ericaceae‐dominant tundra with a noticeable abundance of sedges occupied the main area of the island and then dwarf shrubs gradually decreased. Between 6,400 and 4,600 cal year BP, hearth‐grass‐sedge tundra with a significant component of *Lycopodium annotinum* dominated along the coast.

The dominants in the spectra changed occasionally. Ericaceae and Cyperaceae pollen predominated at this zone, on average 35% each other. The percentage of *Lycopodium annotinum* spores was the highest in this zone (up to 66%). On average Apiaceae pollen abundance was 6%, approximately 6,400 cal year BP it started to increase gradually. Poaceae pollen abundance began to rise circa 6,200 cal year BP. Pollen influx was on average 4,450 grains/cm^2^/year.

### 3.2 McDonald Point‐2 (265–175 cm, 4,600–2,400 cal year BP)

The pollen spectra changed dramatically 4,600 cal year BP, when meadows with the highest levels of Apiaceae emerged. Grasslands began to spread around 3,000 cal year BP.

Apiaceae pollen dominated in this zone (up to 85%). Poaceae pollen reached its maximum in the upper part of this zone. Ericaceae pollen percentage and concentration dropped dramatically. In the interval 3,000–2,400 cal year BP (205–175 cm), Poaceae pollen together with Apiaceae dominated, 38% and 46% on average, respectively. Pollen influx was on average 10,330 grains/cm^2^/year in this zone.

### 3.3 McDonald Point‐3 (175–0 cm, 2,400–0 cal year BP)

After a sharp decrease in Poaceae percentage, sedge tundra with some grasses, umbelliferous, and ferns spread on the coast of Shemya Island. *Lycopodium* and dwarf shrubs rarely occurred in these communities.

The Cyperaceae pollen dominated in this zone (on average 69%). Poaceae and Apiaceae pollen percentages were significantly reduced in comparison with the previous zone, 8% and 10% on average. Pollen influx was on average 6,080 grains/cm^2^/year.

## 4 DISCUSSION

We reconstructed the history of coastal vegetation formation on Shemya Island during almost ten thousand years by comparing new pollen analysis and LOI results with the radiocarbon and stable isotopes data from (Savinetsky et al., 2014). In the early stages of coastal vegetation formation (up to 9,100 cal year BP), dwarf shrub communities were widespread near the McDonald Point peat deposit. In general, heathers (Ericaceae) are a common component of a subarctic island's vegetation in an oceanic climate (Charman, 1994). Dwarf shrub communities dominated by crowberry (*Empetrum*) occurred on well‐drained slopes up to the vegetation border and in the lowlands open to winds in the Aleutian Islands and on the Alaska Peninsula (Heusser, 1978, 1983; Jordan & Krumhardt, 2003). In the Alpine lands, grasses and sedges dominate together with crowberry, and *Lycopodium* dominates with *Empetrum* in the open lowlands (Heusser, 1978). Heusser (1990) highlighted that in the pollen spectra, heathers are an important indicator of habitats which are exposed to strong wind, which has also been noted for Ericaceae in other regions (Breslina, 1987). However, the climate was warm and humid at the Early Holocene (Peteet et al., 2019), which was reflected in the fact that wet sedge tundra predominated in the Shemya interior (Smyshlyaeva et al., 2021) unlike the coastal vegetation with dwarf shrubs (Figures 3 and 4). Island interior was similar to the primary herbaceous vegetation at Atka and Umnak, where the peat profiles were sampled in more sheltered habitats (Heusser, 1973, 1990). Thus, in this study, we have the opportunity to reconstruct vegetation history especially on the coast, not just the entire island or the whole region.

Diatom analysis indicates that relatively deep fresh water existed nearby McDonald Point during the initial stages of peat formation (Neplukhina et al., 2018). However, we only found single *Typha* pollen and *Equisetum* spores in the lowest pollen assemblages (Figures 3 and 4). These taxa are indicators of open water bodies (Galka et al., 2018), but their presence was too insignificant to indicate waterlogged vegetation in the McDonald Point pollen spectra.

The discovery of *Betula*, *Alnus*, and *Pinus* pollen can be attributed to the long‐distance air mass transport from Kamchatka (Heusser, 1990; Noguchi et al., 2018). Thus, we did not consider their variation in the reconstruction of the Aleutian Islands vegetation.

About 9,100 cal year BP, the coastal environment became relatively stable. In general, heather–sedge tundra dominated (Figure 3). The combination of Poaceae, Cyperaceae, and *Empetrum* in the vegetation composition is also typical for the coastal tundra of Alaska (Jordan & Krumhardt, 2003) and other oceanic islands including Shetland, Faroe, Iceland, and the White Sea Islands (Breslina, 1987; Hulme & Shirriffs, 1994; Lawson et al., 2008). There is evidence that at the Early and Early‐Mid Holocene the temperature on the western islands was also colder than on the eastern ones, as in modern times, due to cold air masses from the northwest (Rodionov et al., 2007). However, even during the warmest periods of the Holocene, the sedge‐tundra vegetation prevailed on the Shemya instead of meadows (Figures 3 and 4; Smyshlyaeva et al., 2021).

The relatively drier conditions in the eastern North Pacific circa 8,700 cal year BP (Peteet et al., 2019) were not reflected in noticeable changes in the pollen spectra of McDonald Point. However, the humidity on the western and eastern islands did not change synchronously, as proposed in modeling studies (Rodionov et al., 2005, 2007). The asynchrony of some atmospheric phases along the archipelago was revealed from short‐term observation series, but there are no data on how exactly they were interrelated during the Holocene.

To detect detailed changes in the Shemya seashore environment, we compared results of pollen analysis with δ^15^N values from the same peat profile (Savinetsky et al., 2014). For coastal ecosystems, an increase in this value means the transfer of organic matter from the sea to terrestrial communities (Anderson & Polis, 1999; Caut et al., 2012; Croll et al., 2005; Maron et al., 2006; Szpak et al., 2012). Until 7,100 cal year BP, the δ^15^N averaged 0.12‰, which is typical for arctic and subarctic peatlands (Croll et al., 2005; Davidson et al., 2018; Gąsiorowski & Sienkiewicz, 2019). This value was also close to the values in the interior peat deposit on Shemya Island, which averaged −0.78‰ over the same period of time (Savinetsky et al., 2014). The heavy nitrogen isotope abundance in the McDonald Point peat deposit increased significantly circa 7,100 cal year BP (up to 6.55‰), then about 6,300 cal year BP, it was 4.70‰, and from 5,000 cal year BP, it has steadily increased to 8.42‰ and above (Figures 3 and 4; Savinetsky et al., 2014). There are no δ^15^N measures in the samples 7,100–6,300 cal year BP (310–300 cm) and 6,300–5,000 cal year BP (296–275 cm) due to insignificant amount of organic matter at this part of the core, which is mainly sand or peaty sand (Figures 3 and 4). At this time, there was dry coastal shrub tundra around, and the LOI in the samples was the lowest (Figures 3 and 4). Drier conditions may be related to the next decrease in available moisture around 6,500 years ago (Peteet et al., 2019). However, it could also be due to abrupt changes in sea level, which was established at present altitude by 5,000 years ago (Black, 1980; Kiseleva et al., 2002). As a result of several significant shifts in the coastline, the McDonald Point deposit was covered with sand several times and the peat began to accumulate again, while δ^15^N dynamics remained unknown.

About 4,600 cal year BP, the McDonald Point pollen spectra dramatically changed (Figures 3, 4) from coastal tundra to meadow vegetation with the highest Apiaceae abundance. Higher copresence of Apiaceae and Poaceae in the spectra (Figures 3 and 4) indicates the typical coastal vegetation of the Aleutian Islands and Alaska (Hulten, 1968). These are tall grasses that do not occur on high slopes and places exposed to the wind (Garroutte & Ickert‐Bond, 2013; Heusser, 1990). LOI also increased strongly as a result of the more plant biomass accumulation and/or a decreased degradation rate under lower temperature (Figures 3 and 4). The expansion of tall‐grass meadows would rather take place in the Early Holocene, when the highest temperatures were observed (Peteet et al., 2019) or in the Holocene maximum 7,000–5,000 years ago (Kaufman et al., 2016). However, according to various estimates, circa 4,500 years ago and later, the early Neoglacial events began, which resulted in an increase in storm activity and a decrease in temperature throughout the Bering Sea region (Barclay et al., 2009; Broadman et al., 2020; Kaufman et al., 2016; Peteet et al., 2019). Therefore, it remains unknown what climatic shifts during the subsequent cooling period could lead to the replacement of coastal tundra with more productive meadow vegetation.

At the same time circa 4,600 cal year BP, δ^15^N increased significantly to 12.24‰ in the coastal peat core (Figures 3, 4). In the Arctic ecosystems, the usual nitrogen value does not exceed 3‰ (Janbu et al., 2011; Perren et al., 2012). In many studies devoted to the investigation of island ecosystems, the increment in the heavy nitrogen isotope content in various sediments and organisms is associated with the appearance of seabird colonies and guano fertilization of substrate (Croll et al., 2005; Maron et al., 2006; Szpak et al., 2012). (Caut et al., 2012; Spazk et al., 2012). Similar increases of several ppm are also shown in Svalbard and Greenland over long periods of time or even the entire Holocene (Davidson et al., 2018; Gąsiorowski & Sienkiewicz, 2019; Yuan et al., 2010). In Alaska, fluctuations in the range of 2–3‰ are estimated for fluctuations in the number of sockeye salmon over the millennia, which also reflect the additional input of nutrients from marine ecosystems to terrestrial (Finney et al., 2002). On the Aleutian Islands, higher δ^15^N values were found on islands where large seabirds colonies had survived, compared to islands where Arctic foxes had been introduced, which have subsequently reduced colonies in the past two centuries (Byrd, 1984; Croll et al., 2005; Maron et al., 2006). Thus, we can estimate the seabird impact dynamics on the coastal communities of Shemya Island by heavy nitrogen isotope evaluation.

Vegetation, as one of the components of ecosystems, is also affected by guano fertilization. A study of seabird colonies on the Pacific coast shows that even a short exposure of guano can lead to changes in the soil, plant communities, and chemical alterations in bedrock (Ellis, 2005; Ivanov, 2013). In the absence of human and terrestrial predators, birds can inhabit the entire island and form special ornithogenic ecosystems. Under the bird colonies pressure, peat soils have lower ash content and are enriched with nitrogen and phosphorus (Pleshchenko, 1992). LOI (average 75%) and δ15N (average 13.7‰) in the McDonald Point peat deposit, which differ from the previous values, indicate this impact. This could also lead to changes in the vegetation cover. In general, studies point out a high mosaicity of vegetation and a noticeable change in the dominant species under the influence of bird colonies (Breslina, 1987; Garroutte et al., 2018; Ivanov, 2013). Therefore, we suppose that the input of additional organic matter led to the gradual formation of meadow vegetation on the coast of Shemya Island (Figure 3). According to Breslina (1987), Apiaceae, and Poaceae taxa are typical in the vicinity of bird colonies in the White Sea Islands. To the west of the Aleutians, in the Commander Islands, crowberry gradually decreased following the appearance of bird colonies (Mochalova, 2008) as well as in the McDonald Point pollen spectra (Figure 3). Grasses are significant members of ornithogenic communities (Mochalova, 2008). *Calamagrostis purpurea* and *Leymus mollis* are typical for coastal meadows, but due to avian impact, they grow higher on the slopes and occupy greater areas together with umbelliferous species (Mochalova, 2008). It is important to note that there are no specific species on the Aleutian Islands that would reflect only ornithogenic vegetation, since the islands have impoverished flora due to their remoteness and the considerable length of the archipelago (Garroutte & Ickert‐Bond, 2013; Hulten, 1968; Maron et al., 2006; Mochalova, 2008). We nevertheless found higher diversity of nonarboreal in the pollen spectra during this time (see Data Availability Statement). Pollen influx in McDonald Point deposit also increased (Figures 3 and 4).

Cooling after the Holocene temperature maximum (Kaufman et al., 2016) may have led to a noticeable increase in the productivity of the Bering Sea (Anderson et al., 2005) and, as a consequence, an expansion of seabird colonies. Similar processes took place, for example, in the Greenland region, where the dynamics of the colony was also studied using δ15N (Davidson et al., 2018). However, on no other Aleutian Islands during the Holocene such pollen spectra and traces of the prolonged influence of seabird colonies were found due to the selection of peat deposits in the interior of the islands. Substantial increases in heavy nitrogen (up to 9.32 and 11.40‰) were found in a peat core on Carlisle Island (Islands of Four Mountains, Aleutian Islands) located in the eastern part of the archipelago (Kuzmicheva et al., 2019). But the peaks lasted within a few decades (the multiproxy resolution allows us to state this) circa 7,000 years ago, and then δ15N decreased and remained on average about 3‰ for almost 5,000 years. There were some increases in the relative abundance of Apiaceae and Poaceae at this time; however, there were no noticeable changes in vegetation (Kuzmicheva et al., 2019). At the same time, there was a shift of plant communities toward drier tundra in the interior of Shemya Island circa 4,700 cal year BP, while δ15N remained here on average 1.5‰ (Savinetsy et al., 2014; Smyshlyaeva et al., 2021). Thus, with the long and strong influence of seabird colonies, significant changes in vegetation occurred, which affected only the coast of Shemya Island.

Circa 3,000 cal year BP the relative abundance of Apiaceae gradually began to decrease, while Poaceae percentage increased in the McDonald Point peat deposit (Figure 3). Dominance of these taxa is typical for modern ornithogenic vegetation on the Commander Islands, where grasses and umbelliferous are predominant around the seabird colonies (Mochalova, 2008). Peat remained uniform in density and LOI was consistently high (Figures 3 and 4); thus, the plant communities remained productive. Between 4,000 and 3,000 years ago, the Neoglacial commenced gradually without a pronounced pattern in the region (Barclay et al., 2009; Harada et al., 2014; Kaufman et al., 2016; Peteet et al., 2019). This climatic event was associated with a decrease in temperatures, high ice cover in the Bering Sea, and an intensification of winter–spring storms (Bailey et al., 2018; Harada et al., 2014; Kaufman et al., 2016; Majewski et al., 2004). More severe conditions have resulted in the gradual spread of grass‐meadows in the place of herb‐meadows on Shemya Island (Figures 3 and 4). Vegetation changes occurred around this time on Attu, Adak, and Umnak, where drier tundra spread (Heusser, 1973, 1978, 1990; Noguchi et al., 2018). Changes in plant communities are also associated with climate in other islands, for example on the Kodiak Island (Peteet et al., 2019), Ireland (Birks & Peglar, 1979; Molloy & O'Connell, 2004), the Kuril Islands (Razjigaeva et al., 2013), the Shetland Islands (Hulme & Shirriffs, 1994), and the Faroe Islands (Lawson et al., 2008).

However, the decline of δ^15^N also began about 3,000 cal year BP. For nearly 600 years, the value decreased from 14.0 to 9.0‰ (Figures 3 and 4). This was still several times higher than the δ^15^N median value of the entire peatland (Savinetsky et al., 2014). Grass meadows with forbs dominated on the Shemya coast between 3,000 and 2,400 cal year BP, but the abundance of ornithogenic vegetation gradually decreased (Figure 3). Pollen influx remained relatively high (Figure 4). Savinetsky et al. (2014) suggested that the migration of humans to Shemya reduced the number of colonial birds which resulted in a decline in guano and, accordingly, δ^15^N. Circa 3,000 years ago, at least one settlement existed on the island, and by 2,000 years ago, there were three additional sites, according to archaeological evidence (Corbett & Loring, 2010). The hypothesis of hunting is supported by numerous bird remains identified in these archaeological sites (Lefevre et al., 2010). There were no sufficiently protected habitats on island which could allow a large seabird colony to survive due to flat and low topography of Shemya (Savinetsky et al., 2014). The gradual decline in the colony population as a result of hunting led to a decrease in avian influence on vegetation over time. Anthropogenic settlement notably influenced the vegetation of other oceanic islands (Bennett et al., 1997; Hannon & Bradshaw, 2000; Roy et al., 2018). Strongly pronounced ornithogenic vegetation formation probably did not form on Carlisle Island due to the fact that humans colonized the eastern part of the Bering Sea earlier and also began to reduce seabird colonies around 7,000 years ago (Kuzmicheva et al., 2019). In this case, we argue that, even without domesticated animals or agriculture in the Aleutian Islands, humans could significantly alter ecosystems during the Holocene.

An alternative hypothesis is that the climate influenced seabird population dynamics, which subsequently led to changes in vegetation. Similar impacts have been observed in different regions, including the Atlantic coast of Canada (Diamond & Devlin, 2003), the Bering (Springer et al., 2007), and the Okhotsk seas (Andreev et al., 2005). Avian reproductive success can depend on the oceanic climate on the decadal scale (Bond, 2011). Additionally, avian reproductive success is influenced by the temperature in the winter–spring period, which was altered during the Neoglacial (Harada et al., 2014; Sydeman et al., 2017).

Forb‐grass meadows were abruptly replaced by sedge tundra circa 2,400 cal year BP (Figure 3). Peat did not change outwardly; however, LOI began to fluctuate sharply decreased, and pollen influx also became lower on average (Figure 3). On the coast of Shemya Island, the vegetation did not shift back exactly to dwarf shrub tundra, as it was described for ornithogenic vegetation on other islands with reduced seabird colonies (Breslina, 1987; Ivanov, 2013). On treeless islands, dwarf shrub communities are the final stages of the ecological succession series (Breslina, 1965). Similar communities have been noted on the Kamchatka and Sakhalin Peninsulas (Breslina, 1965). On North Pacific islands, near areas abandoned by seabird colonies more than 10 years ago, tundra with the *Empetrum* dominance formed, thus completing the cycle of vegetation restoration (Ivanov, 2013). Nevertheless, tundra replaced meadows on the Shemya coast, as in studies of modern vegetation in other Aleutian Islands, where the number of birds decreased after the introduction of foxes (Maron et al., 2006). The interior part of the island was not affected by the seabird guano, and, on the contrary, there was a shift to low‐lying dwarf shrub tundra circa 3,600 cal year BP (Smyshlyaeva et al., 2021). Probably, the long‐term nutrients input by avians in the oceanic island environment may continue to influence coastal vegetation even after the reduction of colonies.

From 2,400 cal year BP until recently, δ15N averaged 2.76‰ in the McDonald Point peat deposit (Savinetsky et al., 2014). This corresponds to the values at the Arctic and Subarctic peatlands (Davidson et al., 2018; Yuan et al., 2010), soils of the Aleutian Islands with introduced foxes (Maron et al., 2006), and is slightly higher than in the peat in the Shemya interior, where during the same time the average was 0.81‰ (Savinetsky et al., 2014). The most observable shifts in the coastal pollen spectra occurred when δ15N values passed through 9–10‰ with the presence of a pronounced trend (Savinetsky et al., 2014). If the increase continued, Apiaceae and grasses began to predominate in plant communities, and a decrease in δ15N led to a transition to less productive vegetation (Figures 3 and 4). Moreover, the changes in the heavy nitrogen isotope content had to be long‐term in order to be reflected in the pollen spectra. On Carlisle Island, for example, δ15N was rising above 9–10‰ when bird colonies returned to the island after major eruptions, but both of these increases lasted no more than 100–200 years and had insignificant impact on vegetation (Kuzmicheva et al., 2019). There plant communities also did not return to dwarf shrubs predominance after the decrease in fertilization, as in Shemya. Apparently, the vegetation on the Aleutian Islands after the reduction of seabird colonies, become less productive, but this is not always a shift from productive grasslands to low‐lying dwarf shrubs communities.

Volcanic eruptions also played a significant role in the Aleutian Islands vegetation history (Heusser, 1990; Kuzmicheva et al., 2019; Talbot, Talbot, et al., 2010). Ash falls of various intensities could lead to the complete destruction of island landscape, a change in vegetation dominants, various disturbances of whole plants or their parts, a change in productivity or biomass, or have no effect (Heusser, 1990; Kuzmicheva et al., 2019; Talbot, Schofield, et al., 2010). However, on Shemya Island volcanism did not significantly affect the vegetation as the nearest volcanoes which were active in the Holocene are located 100–200 km to the east (Corbett et al., 2010; Miller et al., 1998); furthermore, there are no ash layers in soil and peat deposits on this island (Kiseleva et al., 2002).

## 5 CONCLUSIONS

Despite the high bird population in the Aleutian Islands, the impact of guano has not been studied in the long term. In order to study the influence of seabird colonies on vegetation, we carried out a pollen analysis of a coastal peat deposit from Shemya Island. About 9,100 cal year BP, coastal vegetation was more or less stable. Sedge–heather tundra spread. The noticeable influence of seabird colonies increased 4,600 cal year BP, when the role of ornithogenic indicator taxa in the pollen spectra dramatically rose. Meadows, instead of tundra, spread out on the coast. Circa 3,000 cal year BP, the bird colonies and their influence on the plant communities declined, due either to the settlement of the island by people who hunted birds or to climate change in the region, including a decrease in average temperatures and increased storminess in spring and winter. The vegetation remains ornithogenic; umbelliferous and grasses dominate in it. Shifts in atmospheric patterns could affect vegetation both directly and indirectly through their impact on bird populations. After a decrease δ15N below 9 ‰ 2,400 cal year BP, the vegetation is replaced by a less productive sedge tundra. This habitat was exposed, but dwarf shrub tundra does not spread on it even after thousand years, as was found on some other Aleutian Islands, where the pressure of the avian influence decreased during the centuries. It is already clear that guano is one of the leading factors in the vegetation formation on the North Pacific islands; however, more studies with better resolution are needed on the other Aleutian Islands to distinguish the key δ15N values which were leading to marked changes in island ecosystems and spatial resolution of this process.

## CONFLICT OF INTEREST

None declared.

## AUTHOR CONTRIBUTIONS

**Olesya I. Smyshlyaeva:** Visualization (lead); Writing‐original draft (lead); Writing‐review & editing (lead). **Elena Erastovna Severova:** Investigation (lead); Writing‐review & editing (equal). **Olga Aleksandrovna Krylovich:** Visualization (supporting); Writing‐review & editing (equal). **Evgeniya Andreevna Kuzmicheva:** Writing‐review & editing (equal). **Arkady Borisovich Savinetsky:** Conceptualization (lead); Funding acquisition (equal); Supervision (lead); Writing‐review & editing (equal). **West Dixie:** Conceptualization (equal); Funding acquisition (equal); Writing‐review & editing (equal). **Virginia Hatfield:** Conceptualization (equal); Funding acquisition (equal); Writing‐review & editing (equal).

## Data Availability

List of pollen samples; pollen count spreadsheet; pollen concentration spreadsheet; stable isotope, LOI and pollen influx results; complete pollen percentage and concentration diagrams: Dryad https://doi.org/10.5061/dryad.0rxwdbs1f.
